# The Cost of Annual versus Biannual Community-Directed Treatment of Onchocerciasis with Ivermectin: Ghana as a Case Study

**DOI:** 10.1371/journal.pntd.0002452

**Published:** 2013-09-19

**Authors:** Hugo C. Turner, Mike Y. Osei-Atweneboana, Martin Walker, Edward J. Tettevi, Thomas S. Churcher, Odame Asiedu, Nana-Kwadwo Biritwum, María-Gloria Basáñez

**Affiliations:** 1 Department of Infectious Disease Epidemiology, School of Public Health, Faculty of Medicine (St. Mary's Campus), Imperial College London, Norfolk Place, London, United Kingdom; 2 Council for Scientific and Industrial Research, Water Research Institute, Accra, Ghana; 3 Neglected Tropical Diseases Control Programme, Disease Control and Prevention Department, Ghana Health Service, Accra, Ghana; University of Ghana, Ghana

## Abstract

**Background:**

It has been proposed that switching from annual to biannual (twice yearly) mass community-directed treatment with ivermectin (CDTI) might improve the chances of onchocerciasis elimination in some African foci. However, historically, relatively few communities have received biannual treatments in Africa, and there are no cost data associated with increasing ivermectin treatment frequency at a large scale. Collecting cost data is essential for conducting economic evaluations of control programmes. Some countries, such as Ghana, have adopted a biannual treatment strategy in selected districts. We undertook a study to estimate the costs associated with annual and biannual CDTI in Ghana.

**Methodology:**

The study was conducted in the Brong-Ahafo and Northern regions of Ghana. Data collection was organized at the national, regional, district, sub-district and community levels, and involved interviewing key personnel and scrutinizing national records. Data were collected in four districts; one in which treatment is delivered annually, two in which it is delivered biannually, and one where treatment takes place biannually in some communities and annually in others. Both financial and economic costs were collected from the health care provider's perspective.

**Principal Findings:**

The estimated cost of treating annually was US Dollars (USD) 0.45 per person including the value of time donated by the community drug distributors (which was estimated at USD 0.05 per person per treatment round). The cost of CDTI was approximately 50–60% higher in those districts where treatment was biannual than in those where it was annual. Large-scale mass biannual treatment was reported as being well received and considered sustainable.

**Conclusions/Significance:**

This study provides rigorous evidence of the different costs associated with annual and biannual CDTI in Ghana which can be used to inform an economic evaluation of the debate on the optimal treatment frequency required to control (or eliminate) onchocerciasis in Africa.

## Author Summary

The African Programme for Onchocerciasis Control (APOC) has recently been extended until 2025, with renewed commitment towards onchocerciasis elimination. This aim is aligned with the goals stated by the World Health Organization and the London Declaration on Neglected Tropical Diseases in January 2012. Switching from annual to biannual (twice yearly) ivermectin distribution might increase the feasibility of onchocerciasis elimination in some African foci. However, relatively few communities have received biannual treatments in Africa, and there are no cost data associated with increasing ivermectin treatment frequency at a large scale, essential pre-requisites to provide reliable information for evidence-based decision making regarding adoption of a biannual treatment strategy. Therefore, we undertook a study to estimate costs associated with biannual compared to annual ivermectin delivery in Ghana, which since 2009 has implemented a biannual treatment strategy in selected priority areas. Our results indicate that the cost of biannual ivermectin treatment per year is approximately 60% higher than the cost of annual treatment. This study provides tangible evidence of the different costs associated with annual and biannual ivermectin treatment, which can be used to inform economic evaluations and policy decisions regarding the optimal treatment frequency required to eliminate onchocerciasis in Africa.

## Introduction

Human onchocerciasis or river blindness is a neglected tropical disease (NTD) caused by the parasitic filarial nematode *Onchocerca volvulus* and transmitted by the bites of *Simulium* blackflies [1]. In addition to ocular pathology (vision loss, blindness), and increased host mortality [2], [3], onchocerciasis also causes disfiguring skin lesions and severe dermal itching that can drastically impair an individual's quality of life [4]. In 1987, ivermectin was registered for human use against onchocerciasis, and Merck & Co., Inc. took the unprecedented decision to donate ivermectin for as long as needed to eliminate onchocerciasis as a public health problem [5].

Two major onchocerciasis control programmes have been launched in Africa. The former was the Onchocerciasis Control Programme in West Africa (OCP), which started in 1974 and closed in 2002, and was initially based solely on vector control until ivermectin was licensed for human use in 1987. For the most part, the OCP used an annual treatment strategy (alone or in combination with antivectorial measures), but in the Western extension, some foci were treated biannually in the absence of vector control [6], [7]. Currently, the former OCP countries undertake their own national onchocerciasis control programmes. The African Programme for Onchocerciasis control (APOC) was launched in 1995 and it has recently been extended to 2025 [8]. It targets the 19 onchocerciasis endemic countries in Africa that were not covered by the OCP (though three of them, Kenya, Rwanda, and Mozambique, were found not to be endemic) [9]. APOC's predominant strategy involves annual, community-directed treatment with ivermectin (CDTI) in areas where the prevalence of onchocercal nodules is greater than 20%, for all those aged five years and older (excluding pregnant or breastfeeding women in the first week after delivery) [9], [10].

Based on the experience in Uganda [11], and the success achieved in most onchocerciasis foci in the Americas [12], there have been recent discussions of switching to biannual treatments (twice yearly) to increase the feasibility of elimination. In the past, only a small number of foci within the OCP (such as River Gambia in Senegal [7]) have received biannual treatment in Africa, and therefore there are no ground-truth data on the cost associated with increasing the treatment frequency to twice per year on a large scale. (In Uganda, the cost of biannual CDTI was simply estimated by doubling that of the annual treatment [11].) Motivated by ivermectin efficacy studies suggesting sub-optimal responses of *O. volvulus* to the drug [13], [14], [15], Ghana (an ex-OCP country), has recently adopted a biannual treatment strategy at a large scale [15].

In Ghana, onchocerciasis is endemic in 9 out of 10 regions with a total at-risk population of approximately 3.2 million [16]. Responsibility for ivermectin distribution—which occurs in 73 districts—was devolved from the OCP to Ghana in 2002 (under the supervision of APOC). Since 2006, onchocerciasis control has been implemented in the context of the Neglected Tropical Diseases Programme (NTDP) [16], and in 2009, 40 (55%) districts started using a biannual ivermectin distribution strategy. The decision regarding which areas should change to the biannual treatment strategy was based on the combined results of rapid epidemiological mapping of onchocerciasis (REMO) conducted in Ghana in 2009, parasitological evaluation via skin snipping and determination of microfilarial prevalence, and entomological evaluations (according to unpublished results of the Ghana onchocerciasis mapping exercise conducted in 2009, and the REMO report summarised in 2010). Areas with an infection prevalence in the adults above 20%, were allocated to a biannual treatment frequency considering also a buffer zone of 20 Km around these CDTI priority areas. Therefore, NTDP decisions as to whether to allocate districts to annual or biannual CDTI were not made on a priori criteria of associated costs but only based on transmission criteria.

In this paper, we report the results of a study undertaken to estimate the costs associated with annual (the standard strategy) vs. biannual CDTI (the newly adopted strategy) in Ghana. We also assess some factors that may hamper the scaling up of treatment frequency at a large scale given that other countries in the region may consider switching from annual to biannual ivermectin distribution.

## Methods

### Ethics Statement

Ethical approval for the study in Ghana was obtained from Imperial College Research Ethics Committee (ICREC) and the Ethics Review Committee (ERC) of the Ghana Health Service (GHS).

### Description of Study Areas

**Figure 1:**
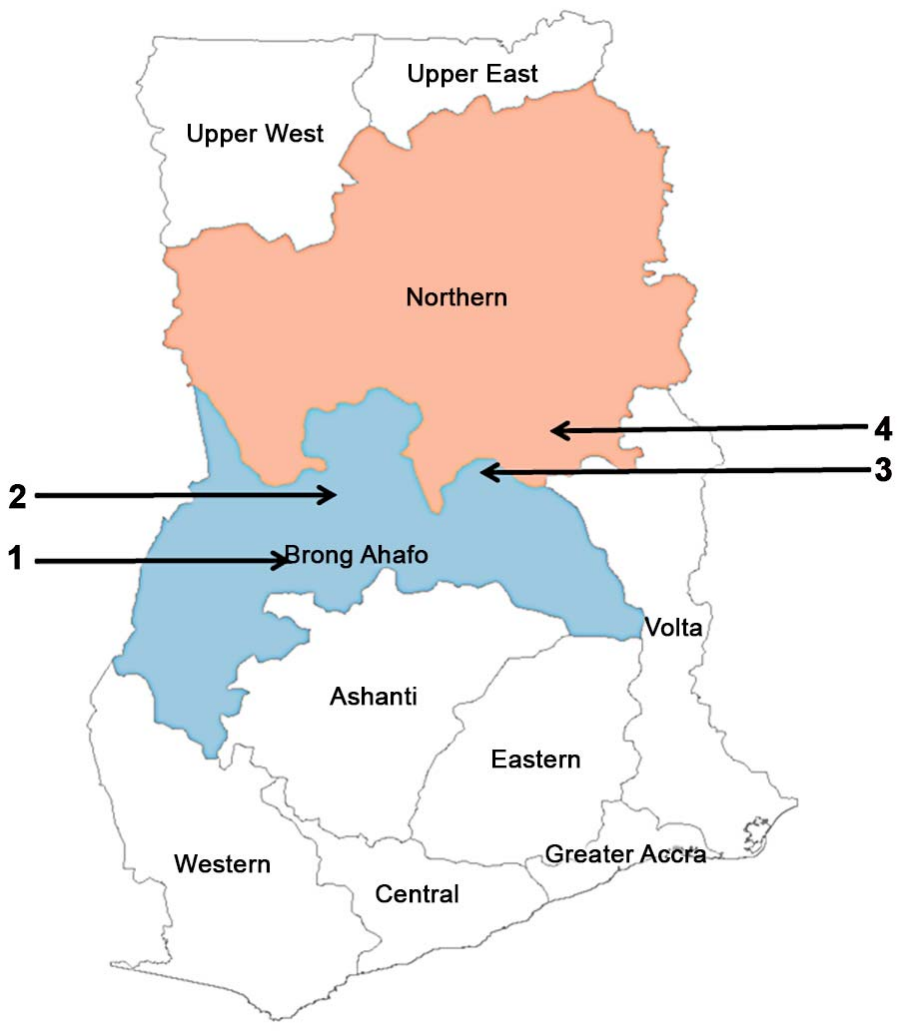
Map of Ghana indicating the sampled regions and districts. The Brong-Ahafo and Northern regions are highlighted in light blue and light pink respectively. 1-Wenchi, 2-Kintampo North, 3-Pru, 4- Kpandai. Figure prepared by Mr Simon O'Hanlon (Imperial College London).

The study focused on the Brong-Ahafo and Northern regions in Ghana. In the former, data were collected in the Wenchi district where CDTI takes place annually; the Pru district and the Kintampo North district, where CDTI is taking place biannually, and in the latter, data were also collected in the Kpandai district, where a mixed strategy (some communities being treated annually and others biannually) is used (Table 1). These districts were selected partly based on logistics at the time of the study, and partly because already established relationships with the GHS at the district and sub-district levels would ensure collection of accurate data via the purposely designed questionnaires (see below). Figure 1 shows the locations of the districts where the study was conducted. As stated earlier, decisions to switch to a biannual treatment frequency were based on infection and transmission criteria alone, so there were no obvious reasons why the decision to change treatment frequency would have been influenced by the local district-specific programme cost.

**Table 1:** Description of ivermectin treatment in the areas where cost data were obtained in Ghana.

Region	District	Treatment Frequency	Number of Persons Treated Per Year	Overall Therapeutic Coverage (%)^a^	Size (km^2^)
Brong-Ahafo	Wenchi	Annual in all communities	27,881	90.43	3,494
Brong-Ahafo	Kintampo North	Biannual in all communities	57,802	82.10	5,108
Brong-Ahafo	Pru	Biannual in all communities	68,506	88.08	2,195
Northern	Kpandai	Annual in 122 (55%) and biannual in 100 (45%) of 222 communities^b^	90,183	79.10	1,772

a For the Wenchi and Kpandai districts, therapeutic coverage estimates were taken directly from national records pertaining to the last treatment round of 2010. For the Pru and Kintampo North districts, coverage estimates were derived from an average of two treatment rounds (the last round of 2010 and the first round of 2011).

b A biannual strategy is used in 15 of 76 (20%) communities in the sampled sub-district, whereas the remainder 80% receive treatment annually. Therefore, the costs are likely to reflect more closely those of annual rather than biannual distribution.

Data were collected at various levels in the organization of the GHS. Firstly, information was gathered by conducting semi-structured interviews at the headquarters of the NTDP in Accra, and at the Regional Health Service directorates in the Brong-Ahafo region. Secondly, districts (and sub-districts where appropriate) were chosen to represent a range of geographical sizes, and population densities (Table 1). Thirdly, community drug distributors (CDDs) were interviewed in at least three communities in each district.

### Perspective

In this study, the costs under investigation were those borne by the health care providers (such as the GHS, the major in-country partners, and the local communities). Therefore the cost of drug manufacture and transport to Ghana were excluded. Only data on the cost of CDTI were collected; costs associated with individual, clinic-based treatment with ivermectin were ignored.

Data were collected on both the financial and economic costs of CDTI. Financial costs are those where a monetary transaction has taken place for the purchase of a resource. Economic costs also include, in addition to the financial costs described above, estimates of the monetary value of goods or services for which no financial transaction has taken place. Therefore, economic costs also account for the value of goods or services which could have been used for another purpose (opportunity costs). Examples of resources which have no financial costs but do have important economic costs are the ‘free’ use of building space provided by the Ghana Ministry of Health, the use of donated vehicles, and the time devoted to CDTI by unpaid CDDs. The costs associated with CDTI arise from various programmatic activities as outlined in Box 1.

**Box 1. Programmatic Activities (partly based on [17])**

- **Drug Distribution Chain:** the process of getting the drugs from where they entered the country to the target population
- **Mobilization and Sensitization:** promotion, information dissemination and advocacy related to the project
- **Training of Volunteers:** training of community drug distributors (CDDs) (includes the costs incurred by both the trainers and the trainees)
- **Other Training:** all other training at whatever level (includes the costs incurred by both trainers and trainees)
- **Reporting:** the preparation and transmission of reports
- **Surveillance and Evaluation:** surveillance of the disease and treatment distribution at all levels
- **All Other Administration:** all other general office administration
- **Other Project Activities:** all other activities not already mentioned above

### Data Collection

Data collection was organized at the national, regional, district, sub-district and community levels and involved interviewing key personnel and scrutinizing national records. Data collected at the national level included records of funds provided by non-governmental organizations (NGO) such as Sightsavers (http://www.sightsavers.org/), and others such as APOC (managed by the World Bank and implemented by the World Health Organization) (http://www.who.int/apoc/en/), among others. Given these multiple sources, it would have been most interesting to obtain a detailed breakdown of the relative contribution of each organization to the funding of onchocerciasis control in Ghana. Unfortunately, even at the national sampling level, it was rarely possible to separate the costs by their funding source. This, however, did not affect the study, which focused on the aggregate cost of onchocerciasis control. The costs collected were incurred in the year 2011. At each level, costs were collected according to different resource types (Box 2) using an approach based on methods described by McFarland *et al*. [17] and the UNAIDS guidelines for costing studies [18]. First, the total gross expenditure on a resource (per year) was calculated from national records and/or questionnaires. Second, the most appropriate person(s) to answer questions on how the resource is used for activities relating to onchocerciasis control was selected for interview. Third, the interviewee was asked to indicate what fraction of time the resource was used for onchocerciasis control over the year (this was corroborated by multiple sources where possible). Multiplication of the total gross cost and fraction of time attributable to onchocerciasis control yielded an estimate of the recurrent yearly cost for a resource (such as an employee). The cost of capital resources—goods that last for more than one year, such as cars and computers—were estimated in a similar fashion, but the gross cost was spread over the average useful lifetime of the resource (a technique known as ‘annualization’) to arrive at an average yearly cost [18]. (An annualization and discount rate of 3% was used to calculate the economic costs of capital resources [19].) The average useful lifetime of all capital goods was assumed to be five years, in line with the value estimated by McFarland *et al*. [17] and corroborated by study participants at the national level. However, the sensitivity of the results to this assumption was investigated by varying the average useful lifetime between 5 and 8 years [20]. The annual cost of building space was estimated as the equivalent market rental value for the space being used for the control programme [18].

**Box 2. Resource Types (partly based on [17])**

- **Transportation (Capital Costs):** the capital costs associated with vehicles (e.g. the annualized^a^ cost of motorbikes and cars)
- **Transportation (Recurrent Costs):** the recurrent costs associated with transport (e.g. fuel, insurance, maintenance, repairs, and rental costs)
- **Personnel:** the recurrent costs associated with paying salaries to employees (including any supplements or other benefits of employment)
- ***Per Diems*** **:** the recurrent costs associated with daily allowances (*per diems*)
- **Supplies and Equipment (Capital Costs):** other capital costs associated with a project, (e.g. annualizeda costs of computers, photocopiers, and generators etc.)
- **Supplies and Equipment (Recurrent Costs):** the recurrent costs associated with project activities and general office running
- **Overheads:** the recurrent indirect costs associated with a project's specific utilities charges, building rental or equivalent
- **Volunteer Community Drug Distributor (CDD) Time:** the monetary value of the donated time of CDDs and other community members in implementing community directed treatment with ivermectin (CDTI)

**^a^** The annual share of the initial cost of capital equipment

The interviewee was also asked to estimate the fraction of time that the resource was used for itemized onchocerciasis control programmatic activities (Box 1). In addition, in districts receiving ivermectin biannually, the interviewees were asked to describe how their time spent on different CDTI activities had changed since increasing the treatment frequency to twice per year, and to indicate which of the CDTI activities are repeated for both treatment rounds.

At each level, and where relevant, interviewees were given the opportunity to express whether they had encountered any specific difficulties with the increasing of treatment frequency.

### Data Analysis

**Figure 2:**
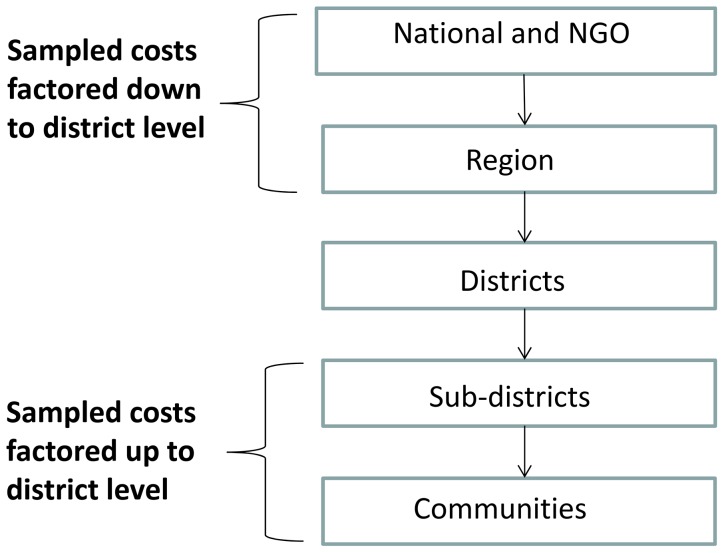
Organization levels at which data on cost of ivermectin distribution were collected.

Costs collected at the national and regional levels, were factored down and costs from the sub-district and community levels factored up, with the aim of arriving at a value for the cost per person treated per year in each district (Figure 2). This is described for each of the levels below.

#### National costs

Of the 73 districts in Ghana where ivermectin is distributed, 40 (55%) are implementing biannual treatment. Consequently, when allocating the national costs to the districts, the costs were weighted according to the district's frequency of treatment. Based on responses to questionnaires, scrutinizing of national records, and conduction of semi-structured interviews, it was estimated that districts treating biannually were responsible for 70% of the total national cost. Separate costs (according to annual or biannual treatment) were allocated equally across districts receiving a certain treatment frequency. Based on interviews at the headquarters of the NTDP and the McFarland *et al.* study [17], it was assumed that the main drivers of the national costs were independent of target population size and therefore we did not adjust the national costs by the size of districts' target populations.

#### Regional costs

These were distributed among districts using the same frequency of treatment-based weighting as used for the national costs. Due to logistic reasons on the terrain, it was only possible to estimate regional costs from one of the two regions from which districts were sampled. Thus, the costs incurred by the Northern region were assumed to be the same as those estimated from data pertaining to the Brong-Ahafo region.

#### Sub-district costs

In each district included in this study one sub-district was sampled. The costs incurred by the sampled sub-districts were multiplied by the number of sub-districts within each district to aggregate the costs to the district level.

#### Community costs

In each district included in this study three communities were sampled. In each sampled community, questionnaires were administered to the CDDs to ascertain to how many people they distributed ivermectin, and whether they received compensation from the district (this was corroborated at the local district health centres). Additionally, the opportunity cost of the volunteer CDDs' donated time was estimated by asking CDDs how much time they spent distributing the drug each treatment round. This donated time was converted to an equivalent number of 8-hour working days, which were valued according to the minimum wage in Ghana in 2011 (3.73 Ghana Cedis (GHC) per day [21]). This figure was reported to be equivalent to the daily wage of a hired farmland worker in the Brong-Ahafo region, the occupation of the majority of the interviewed distributors, and was subsequently used to estimate the value of the CDDs donated time across each district. However, to place a precise value on a CDD's donated time is difficult and whether or not it should be included is a matter of debate. Furthermore, the daily wage of a hired farmland worker can vary from district to district, and especially from region to region [20], [22]. Therefore, we calculated the economic cost both including and excluding CDD's donated time, and investigated the sensitivity of the results to the assumed daily wage (increasing or decreasing it by GHC 1.00).

#### Currency conversion

All costs were converted from the Ghanaian local currency (GHC), to United States dollars (USD), using the average 2011 exchange rate of USD 1.00 to GHC 1.58 [23]. Reported costs from other studies were also converted to 2011 US dollars (using a consumer price index inflation calculator [24]) to allow for valid comparison with our results.

## Results

Table 2 shows the estimated financial and economic costs—including and excluding volunteer CDDs' time—of CDTI in the four sampled districts. The majority of the costs associated with CDTI were financial, with the extra economic cost per person per year (excluding CDDs' time) only adding USD 0.01–USD 0.03 (this includes the value of donated vehicles and use of free building space).

**Table 2:** Financial and economic costs (USD^a^) per person treated per year in each district.

Frequency of CDTI^b^	Annual	Biannual	Biannual	Mixed^c^
Cost type	Wenchi	Kintampo North	Pru	Kpandai
Financial cost	0.39	0.62	0.58	0.40
Economic cost (excluding volunteer CDD's^d^ time)	0.40	0.64	0.60	0.43
Economic cost^e^ (including volunteer CDD's time)	0.45	0.73	0.69	0.50

a USD: US Dollars.

b CDTI: Community-directed treatment with ivermectin.

c Data from Kpandai district reflect a combination of annual (in 61 of 76 (80%) of the communities in the sampled sub-district) and biannual treatment frequency (see Table 1 and main text).

d CDD: Community Drug Distributor.

e Economic costs include financial costs (monetary transactions) and estimates of the monetary value of goods or services for which no financial transaction has taken place (for example, the opportunity cost of the CDDs' time donated to administer ivermectin rather than working their fields) [18].

The estimated economic cost (excluding CDDs' time) of annual treatment in Wenchi district is USD 0.40 per person per year. The economic costs (excluding CDD's time) of biannual treatment in the Pru and Kintampo North districts are approximately 50–60% higher (USD 0.60 and USD 0.64 per person per year respectively) than the corresponding annual costs. The estimated economic cost (excluding CDDs' time) for Kpandai district—which uses a combination of an annual and biannual strategy (see Table 1 for description)—is USD 0.43 per person per year. These results were not sensitive to the assumed average useful lifetime of capital goods; changing this from 5 to 8 years only changed the cost per treatment by a maximum of USD 0.015.

### Costs Disaggregated by Resource Type and Programmatic Activity

**Figure 3:**
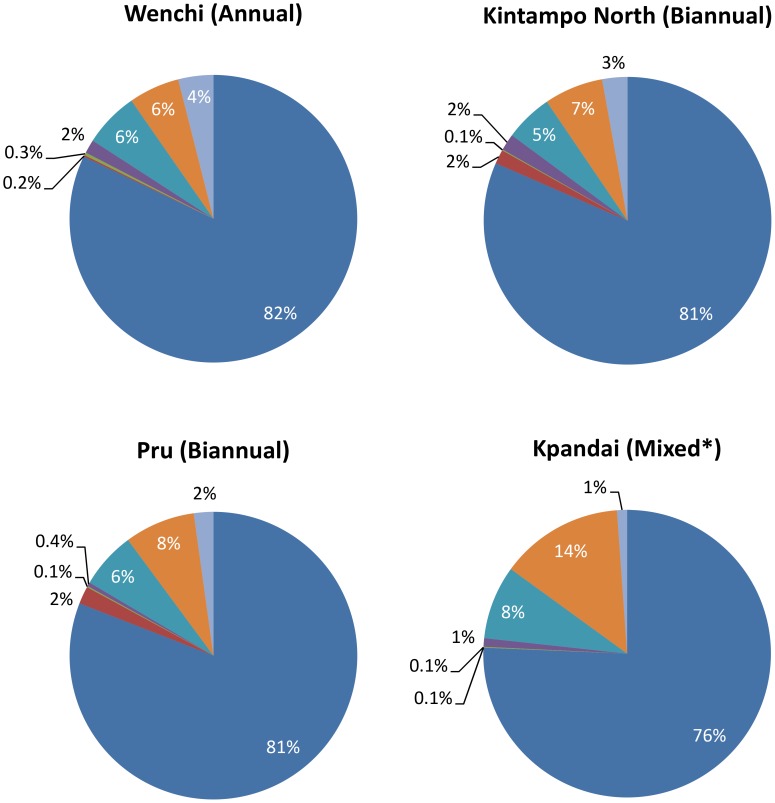
Economic costs at district, sub-district, and community levels disaggregated by resource type (excluding CDDs' time). *Personnel* (dark blue); *Per Diems* (red); *Supplies and Equipment (Capital costs)* (green); *Supplies and Equipment (Recurrent costs)* (purple); *Transportation (Capital costs)* (turquoise blue); *Transportation (Recurrent costs)* (orange); *Overheads* (light blue). Definitions of different resource types are given in Box 2. *Data from Kpandai district reflect a combination of annual and biannual treatments.

Figure 3 depicts the cost of onchocerciasis control by CDTI disaggregated by resource type in the four sampled districts. The largest proportion of the total cost was associated with the payment of personnel. Recurrent transportation costs, such as the costs of fuel and vehicle maintenance, were the next most costly resource and showed the most variation among districts.

**Figure 4:**
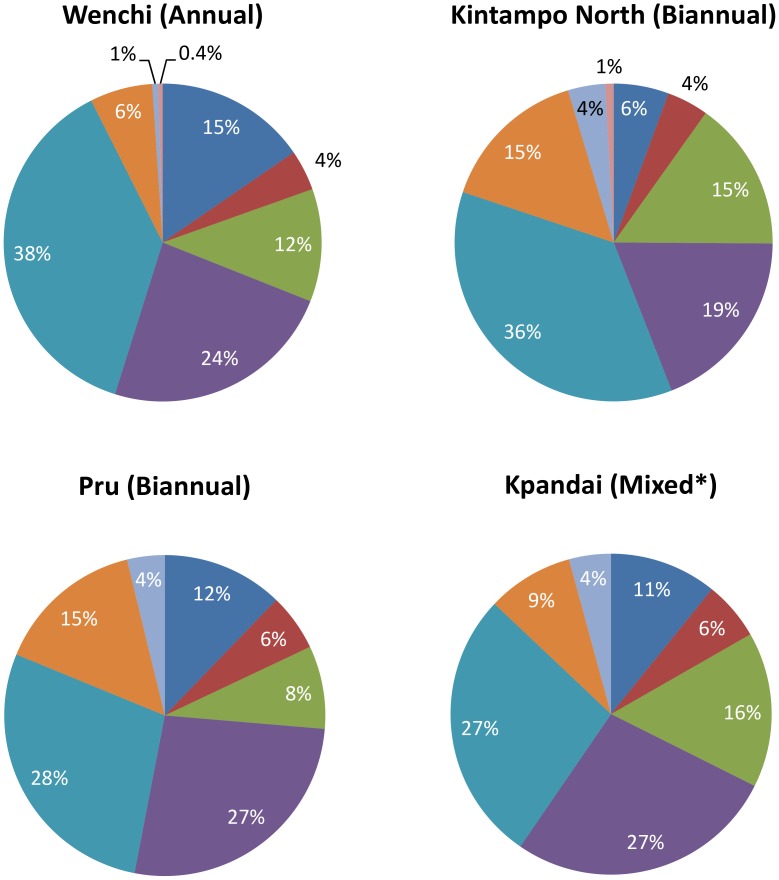
Economic costs at district, sub-district, and community levels disaggregated by programmatic activity (excluding CDDs' time). *Training of Volunteers* (dark blue); *All Other Training* (red); *Mobilization/Sensitization* (green); *Drug Distribution Chain* (purple); *Surveillance and Evaluation* turquoise blue); *Reporting* (orange); *All Other Administration* (light blue); *Other Project Activities* (pink). Definitions of programmatic activities are given in Box 1. *Data from Kpandai district reflect a combination of annual and biannual treatments.

Figure 4 depicts the cost of CDTI-based onchocerciasis control disaggregated by programmatic activity in the four sampled districts. Surveillance and evaluation incurred the highest cost, followed by the drug distribution chain. For Pru and Kintampo North districts, the data show a noticeable increase in the reporting cost compared to Wenchi district.

### Community Distributors

From the pooled community data, it was estimated that there is one CDD for every 390 people and they spend an average of 61 hours distributing ivermectin each treatment round. The above value was used with data on the number treated in each district (Table 1) to estimate the total amount of time CDDs spend distributing the drug across the whole district. This increased the economic cost by USD 0.046 per person per year when treating annually, and by USD 0.092 when treating biannually (Table 2). This result was robust to the assumed daily wage of a hired farmland worker, which when increased or decreased by GHC 1.00, only changed the economic cost of CDD per treatment by plus or minus USD 0.012.

The CDDs reported receiving an average equivalent of USD 3.17 in compensation for attending the distribution training sessions (which are conducted before each treatment round), and between USD 3.17 and USD 9.52 after distributing the drug. In this analysis, it was assumed that each distributor received the average (arithmetic mean) of the reported values (a total of USD 9.96 in compensation for both training and distribution for each treatment round).

### Reported Difficulties

The implementation of a large-scale, mass biannual ivermectin treatment strategy was reported at the district and sub-district level as being well received and perceived as sustainable in the future. However, the disease control officers at the district health centres in the sampled districts in which biannual treatment is being implemented, reported that increasing the treatment frequency to twice per year substantially increased the workload by increasing the amount of time they spent on reporting activities (the percentage of the economic cost at the district, sub-district, and community levels attributed to reporting activities increased from 6% in the districts (Wenchi) treated annually to 15% in the districts treated biannually (Pru and Kintampo North) (Figure 4)).

## Discussion

The estimated economic cost of annual CDTI in Wenchi district, i.e. USD 0.40 per person per year excluding CDDs' time, is consistent with the lower range of costs reported by McFarland *et al*. [17], who estimated an average economic cost (excluding CDDs' time) of USD 0.62 (2011 prices) per person per year from 10 regions (excluding one region co-endemic with *Loa loa*) across Cameroon, Nigeria and Uganda (with values ranging from USD 0.39 to USD 2.77 (2011 prices)). The estimated cost of annual CDTI presented here is 1.4 times higher than the USD 0.29 (2011 prices) per person estimated by Onwujekwe *et al*. [25] using data from two Nigerian communities. However, the Nigerian study used a smaller sample of only two communities, and did not collect costs from as an extensive range of sources as we did here, or as done by McFarland *et al*. [17]. Katabarwa *et al*. [26] estimated that in districts of a similar population size to Wenchi, the cost per treatment was USD 0.34 (2011 prices) [26]. However, in districts with a larger population (>100,000 inhabitants) the cost fell substantially to USD 0.13 (2011 prices) [26]. These estimates are broadly consistent with the cost of annual mass drug administration (MDA) for lymphatic filariasis control presented by Goldman *et al.* [27], in which the estimated financial cost per treatment (with donated ivermectin and albendazole) in Ghana was USD 0.21 (2011 prices) but varied between USD 0.08 and USD 2.91 (2011 prices) across the whole multi-country study.

The estimated cost of biannual CDTI per person per year in the Pru and Kintampo North districts was 50–60% higher than the estimated cost of annual (in Wenchi) treatment. This is consistent with the estimated increase in costs associated with biannual MDA for lymphatic filariasis control provided by Stolk *et al.* [28] (who estimated for Africa, a 63% increase in the cost of treatment per year excluding the value of donated drugs). These costs are higher than estimates for biannual treatment at smaller scales and specific age groups, such as in school-based anthelmintic treatment programmes. For instance, Phommasack *et al*. [29] found that the annual cost of treatment of soil-transmitted helminthiases in a school-based programme was 35% higher in provinces treating biannually than in those treating annually. However, school-based treatment programmes are implemented differently than community-based programmes and therefore the change in costs of different treatment frequencies cannot be directly compared.

Caution is also advised when comparing the costs of different strategies estimated using data from different districts. This is because districts have different characteristics, such as road conditions, spread of communities, and population densities, which will affect the estimated cost of CDTI. Because of these potential difficulties, study participants in the Pru and Kintampo North districts were asked to estimate—based on their previous experience—the percentage allocated to a given resource had this resource hypothetically been used for an annual treatment strategy. Thus, the estimated hypothetical economic cost (Table 3) of treating annually in the Pru and Kintampo North districts (USD 0.39 and USD 0.43 per person per year, respectively) were consistent with the actual cost estimates of treating annually obtained for Wenchi (USD 0.40 per person per year). This supports the estimated 50–60% increase in costs when treating biannually compared to treating annually. The difficulties associated with comparing fairly costs among districts within Ghana exemplify a more general conundrum of comparing results of health economic analyses conducted in different locations, such as the complexity of comparing data collected from different countries with differently structured economies and healthcare systems, and where public health interventions may comprise different (e.g. school-based versus community-based) modalities of delivery.

**Table 3:** Hypothetical cost (USD) of annual CDTI in Kintampo North and Pru districts, Brong-Ahafo region, Ghana.

Cost Type	Estimated Annual Cost Per Person Treated if Annual Distribution were Implemented	
	Kintampo North	Pru
Financial cost	0.42	0.38
Economic cost (excluding volunteer CDD's time)	0.43	0.39
Economic Cost (including volunteer CDD's time)	0.47	0.44

Abbreviations and cost explanations as in Table 2.

Our estimated economic cost of CDTI in the Kpandai district, where both annual and biannual treatments are delivered, likely reflects more closely the cost of annual rather than biannual CDTI since only 15 of 76 (20%) of the communities in the sampled sub-district receive biannual treatment (with the remaining 80% receiving annual CDTI). This possibly explains why the estimated cost per person per year in the Kpandai district was only marginally higher than that in Wenchi (USD 0.43 for the former versus USD 0.40 for the latter), in which only annual treatments are delivered. Furthermore, Kpandai has a very high population density which could reduce the cost per treatment (as found in [26]). Across the whole district, 122 of 222 (55%) of the communities are treated annually and the remaining 45% receive biannual CDTI. Therefore, it is reasonable to expect overall, the actual cost of ivermectin treatment for Kpandai will lie in between the estimated costs of annual and biannual CDTI.

### Costs Disaggregated by Resource Type and Programmatic Activity

The costs disaggregated by resource type were consistent among the sampled districts. These data are also similar to those presented by McFarland *et al*. [17]. The recurrent transportation cost was notably higher in Kpandai compared with the other districts. This may in part be due to the poorer quality of roads in the area, resulting in higher vehicle maintenance and fuel costs (although many other factors, including the spread of the communities, also affect transportation costs). The costs disaggregated by programmatic activity showed slightly more variation among districts than among the different resource types. It is noteworthy that in the Pru and Kintampo North districts (and to a lesser extent in the Kpandai district), the percentage of the economic cost attributed to reporting activities at the district, sub-district, and community levels is substantially higher than that in the Wenchi district (15% in Pru and Kintampo North compared to 6% in Wenchi) (Figure 4). This was attributed to the increase in treatment frequency and is discussed in further detail in the section on *Reported Obstacles Associated with Switching from Annual to Biannual CDTI.*

### Community Distributors

The compensation system for CDDs has recently been implemented in Ghana to cover their transport costs, to facilitate attendance of training days, and to help serve as an added incentive. The amount received by CDDs per treatment round was corroborated at the district health centres. Generally, the reported amount received by the community distributors was very consistent across communities and districts.

Accounting for the volunteer CDDs' time added approximately USD 0.05 per person per treatment round. The is consistent with the value reported by Onwujekwe *et al*. [25], who found that taking into account volunteer CDD time in two Nigerian communities added approximately USD 0.07 (2011 prices) per person per treatment round (using the Nigerian minimum wage to value the volunteer CDDs' time). However, both our and the Onwujekwe *et al.* [25] estimates are substantially lower than that reported by McFarland *et al*. [17], who estimated that accounting for volunteer CDDs' time added an average of USD 0.19 (2011 prices) per treatment round (valuing volunteer time based on the average per capita Gross National Income (GNI) for each of the three countries studied in [17], namely, Cameroon, Nigeria and Uganda). However, this estimate was highly variable between the different study sites (USD 0.05–0.54 (2011 prices) per treatment round). The use of different methods to value donated CDDs' time (see below) could partly explain the difference (i.e. estimation using the country's minimum wage, or using the country's per capita GNI). Other possible explanations include the occurrence of cultural differences affecting the time it takes to distribute the drug.

As mentioned above, the method used to value the volunteer CDD's time has marked effects on the cost output. For example, we assumed the market value of the volunteer CDD's time to be USD 2.36 per day (the minimum wage in Ghana of GHC 3.73 divided by the 1.58 exchange rate [23]) based on the wage that a farmland worker would receive (i.e. the wage received for the most common alternative occupation) [21], [30]). However, had we valued the volunteer CDDs' time using the per capita GNI method (as used by McFarland *et al.* [17]), this figure would have increased to USD 4.96 per day [21], [30]. This difference may seem relatively small but when these costs are factored up to the district level, they can become substantial.

### Reported Obstacles Associated with Switching from Annual to Biannual CDTI

Disease control officers at the district health centres reported that increasing the treatment frequency to twice per year increased substantially the amount of time they spent on reporting activities. This is consistent with the costs disaggregated by programmatic activity (Figure 4), which indicate that the time spent on reporting activities increased more than any other project activity when comparing biannual and annual treatments. This may potentially lead to delays in ivermectin being delivered to the districts, if the necessary reports for the next dispatch of drugs are not completed on time (delivery of the next batch of ivermectin being contingent on reporting). Delays in the delivery of treatment to communities not only will have administrative implications, but more importantly, transmission implications. Treating individuals every 6 months is highly important for transmission suppression, as it has been estimated that adult *O. volvulus* female worms start recovering from the temporary sterilising effects of ivermectin approximately between three and four months after treatment, and by six months microfilarial production has recuperated to a substantial degree [31]. Therefore, delays in treatment will permit more transmission, ultimately making the disease harder to eliminate and diminishing the benefit of treating biannually. National onchocerciasis control programmes which consider increasing CDTI frequency may need to support reporting activities at the district level and potentially at the drug donation programme level to encourage timely reporting but also to allow greater flexibility in deadlines to minimize delays in drug distribution.

### Data Limitations

In Ghana, onchocerciasis control is under the remit of the NTDP and therefore different disease control programmes are often integrated. For example, onchocerciasis and lymphatic filariasis control activities are often carried out simultaneously. Potentially, this can lead to difficulties in obtaining accurate costs for a single disease intervention. In addition, this study was retrospective, and therefore, to a certain extent, the data obtained were subject to some degree of recall bias.

In order to reduce the time and logistical complexity involved in collecting the cost data, our sampling strategy was not random, as we purposely visited local government offices and communities in districts where CDTI was annual, biannual, or a combination of the two. However, we were only able to obtain data in one district that implements annual treatment and one sub-district in each of the districts. Also, the selected districts may have been more accessible by road from Accra, the capital of Ghana, than other more remote locations. Nonetheless, there is no reason to assume that the costs reported for the sites included in this study (either delivering annual or biannual CDTI) are not representative of other sub-districts in the area, nor is there a treatment cost-associated reason as to why an area switched from annual to biannual CDTI other than the parasitological criteria listed above. This is confirmed by the similarity of cost estimation of annual treatment between the districts delivering only annual CDTI and the sub-districts also delivering yearly treatment within districts implementing both strategies. Due to logistic reasons, the regional level costs in the Northern region were assumed to be the same as those estimated from Brong-Ahafo region. However, due to differences between the regions (such as road networks and community scattering), the costs incurred in the Northern region may be higher. Nevertheless, this assumption will not affect the main conclusions of the study regarding the relative costs of annual vs. biannual treatment.

### Concluding Remarks

Our estimate of the cost of annual CDTI is consistent with the range of values previously reported in the literature [17], [25], [26]. Our results indicate that the cost of biannual ivermectin treatment was approximately 50–60% higher than the cost of annual treatment, and that simply doubling the cost of annual CDTI does not yield a correct estimate as some studies have assumed [11]. This is higher than estimates for increasing treatment frequency obtained at smaller scales and when targeting specific age groups, such as those associated with school-based anthelmintic treatment programmes [29], which are not truly relevant for onchocerciasis, but similar to estimates for the more comparable lymphatic filariasis control programme [28]. Our study will be beneficial in informing economic evaluations regarding cost-effectiveness analyses of increasing CDTI frequency from annual to biannual in the African context for the control and elimination of human onchocerciasis.
